# Highly tunable refractive index visible-light metasurface from block copolymer self-assembly

**DOI:** 10.1038/ncomms12911

**Published:** 2016-09-29

**Authors:** Ju Young Kim, Hyowook Kim, Bong Hoon Kim, Taeyong Chang, Joonwon Lim, Hyeong Min Jin, Jeong Ho Mun, Young Joo Choi, Kyungjae Chung, Jonghwa Shin, Shanhui Fan, Sang Ouk Kim

**Affiliations:** 1 Department of Materials Science and Engineering, National Creative Research Initiative Center for Multi-Dimensional Directed Nanoscale Assembly, KAIST, Daejeon 34141, Republic of Korea; 2 Department of Materials Science and Engineering, KAIST, Daejeon 34141, Republic of Korea; 3 Department of Electrical Engineering, Stanford University, Stanford, California 94305, USA

## Abstract

The refractive index of natural transparent materials is limited to 2–3 throughout the visible wavelength range. Wider controllability of the refractive index is desired for novel optical applications such as nanoimaging and integrated photonics. We report that metamaterials consisting of period and symmetry-tunable self-assembled nanopatterns can provide a controllable refractive index medium for a broad wavelength range, including the visible region. Our approach exploits the independent control of permeability and permittivity with nanoscale objects smaller than the skin depth. The precise manipulation of the interobject distance in block copolymer nanopatterns via pattern shrinkage increased the effective refractive index up to 5.10. The effective refractive index remains above 3.0 over more than 1,000 nm wavelength bandwidth. Spatially graded and anisotropic refractive indices are also obtained with the design of transitional and rotational symmetry modification.

## Abstract (web summary)

Wider control of the refractive index is desired for new optical applications. Here the authors manipulate block copolymer self-assembled nanopatterns via shrinkage in order to control the refractive index. They achieve an index above 3 over 1,000 nm bandwidth.

## Graphical abstract

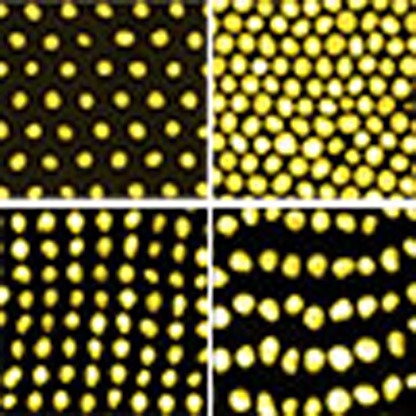

The refractive index (in this work ‘refractive index' indicates the real part of the frequency-dependent complex refractive index, while the imaginary part is referred to as ‘extinction coefficient') is a fundamental optical parameter that governs the emission and absorption as well as the propagation of light in a medium, including the velocity, attenuation, refraction and diffraction. Materials with a high and controllable refractive index are crucial for diverse optical devices ranging from high-resolution imaging and lithography to optical communication systems and light management for optoelectronic devices^1^,^2^,^3^,^4^,^5^. However, the refractive indices of natural transparent materials are an intrinsic property of materials that is difficult to tailor. The ranges of the indices are also limited to below 2*–*3 for visible wavelengths because of the small transition dipole moments of electronic resonances of atoms and molecules^6^,^7^,^8^,^9^.

A potential route to overcome this intrinsic limitation can be offered by visible-range metamaterials^10^,^11^,^12^,^13^,^14^,^15^. High-refractive-index non-resonant metamaterials have been experimentally demonstrated up to the terahertz frequency range^16^,^17^. Scaling up of the operating frequency to the visible range has not been accomplished because of two basic difficulties. First, while a large dielectric constant can be achieved with the use of electric responses of metallic objects, these objects tend to have substantial non-resonant diamagnetic responses^16^. A previous approach to eliminate this diamagnetic response resulted in metallic objects with complex shapes that are very difficult to fabricate in the visible wavelength range^16^,^17^. Second, to construct a metamaterial with desired properties requires detailed control of the symmetry and the lattice period. Bottom-up approaches such as block copolymer (BCP) self-assembly with an innate ability to produce regular arrays with nanoscale periods can be useful for fabricating visible-light metamaterials^18^,^19^,^20^,^21^,^22^. Unfortunately, the possible unit cell shapes and lattice configurations are substantially restricted as the period and symmetry of the self-assembled morphology is fundamentally limited by thermodynamics.

Here we exploit the nanometre-scale resolution of a bottom-up approach to realize a physical principle that allows an unprecedented level of control of the refractive index in the visible and infrared regions. Unlike previously reported visible metamaterials^10^,^23^, our index-control principle does not depend on resonance directly and thus presents small dispersion and low optical loss over a broad bandwidth from radio frequency up to optical frequency away from the resonance frequency. The suggested design is surprisingly simple but functional: a planar array of metal nanoparticles—the exact shape of the nanoparticles is not critical—with spatially and directionally controlled spacing. The desired metal particle array is fabricated by BCP self-assembled nanopatterning on thermal shrinkage films, and subsequent pattern shrinkage allows for period and symmetry control. The shrinkage film is a polymeric substrate with a lateral dimension that is reducible by an entropy-driven mechanism^24^. The fabricated sheet of the nanoparticle array can be considered a homogeneous thin film with an effective relative permeability (*μ*_eff_), permittivity (*ɛ*_eff_) and index (*n*_eff_), as the array period is much smaller than visible wavelengths^25^. With this approach, an *n*_eff_ as high as 5.10 is experimentally confirmed with a large optical anisotropy and a spatial index gradient is demonstrated.

## Results

### Theoretical design of tunable refractive index metamaterials

The physical principle underlying our approach is based on the large difference between the Thomas-Fermi screening length (TFSL)^26^ and the magnetic field penetration length (skin depth) in metals^27^. If the size (*d*) of the metal particles is much larger than TFSL (*d*≫TFSL≈0.5 Å), a sufficient amount of charges can be induced on the particle surface to counterbalance an applied electric field. By contrast, if the particle size is similar or smaller than the skin depth (nearly constant at 20–25 nm for silver and gold in the broad infrared range, with higher values in the visible), magnetically induced eddy currents are small, resulting in a weak magnetic dipole moment inside the particles^28^. Consequently, small metal particles (with TFSL<<*d*≤ skin depth) play the role of meta-atoms with large electric polarizability and negligible magnetic polarizability. We note that interconnected structures can possess unusual optical properties rising from topology- and polarization-dependent resonances^20^,^21^,^22^. On the other hand, the isolated metallic inclusions proposed here allow an easy control on the electric and magnetic responses over a broad frequency range in the long-wavelength regime. In addition, this phenomenon is notably in contrast with most metamaterials with isolated inclusions that have been considered to date, where metallic inclusions are typically thicker than the skin depth (*d*> skin depth), thereby exhibiting considerable magnetic polarizability. Moreover, the electric and magnetic polarizabilities for those large particles are coupled to each other, and it was previously shown that the resulting *ɛ*_eff_ enhancement is almost exactly cancelled by the *μ*_eff_ reduction such that *n*_eff_ [=(*ɛ*_eff_ × *μ*_eff_)^1/2^] remains near unity in the long-wavelength regime (that is, when the wavelength is much larger than the array period and the fundamental resonance wavelength), regardless of the volume fraction of the particle^16^. Therefore, the use of particles smaller than the skin depth is crucial in severing the interdependence of electric and magnetic polarizabilities and in controlling *n*_eff_.

With these electrically polarizable but magnetically inactive particles, *n*_eff_ is easily controllable with the spacing (*g*) between the particles. When *g*<<*d*, a recently proposed homogenization theory states that *ɛ*_eff_ in the long-wavelength regime is enhanced with respect to the permittivity of the gap-filling dielectric (*ɛ*_d_) such that *ɛ*_eff_∼*ɛ*_*d*_*(d+g)*/*g* (refs 16, 17, 27). (The near-field coupling between particles and resulting sensitivity to *g* prevent the prediction of the effective refractive index in our system by well-known particle homogenization methods including the Maxwell-Garnett formalism^29^, the Bruggeman formalism^30^ or their derivatives^31^.) With near-unity *μ*_eff_, the controllability of *n*_eff_ directly follows *n*_eff_∼[*ɛ*_*d*_ *(d+g)*/*g*]^0.5^. Therefore, high *n*_eff_ can be achieved by implementing a large *d/g* ratio^27^. (In this classical model, an arbitrarily large enhancement factor can be obtained if a sufficiently small *g* is utilized, and the ultimate measurable enhancement would be determined by quantum tunnelling and non-local effects of electrons^32^ as well as process uniformity.) Moreover, by preparing anisotropic gap sizes for two different directions (or by inducing spatially graded gap sizes), index anisotropy (or gradient) can be readily obtained.

### Fabrication of visible metamaterials from self-assembly

**Figure 1:**
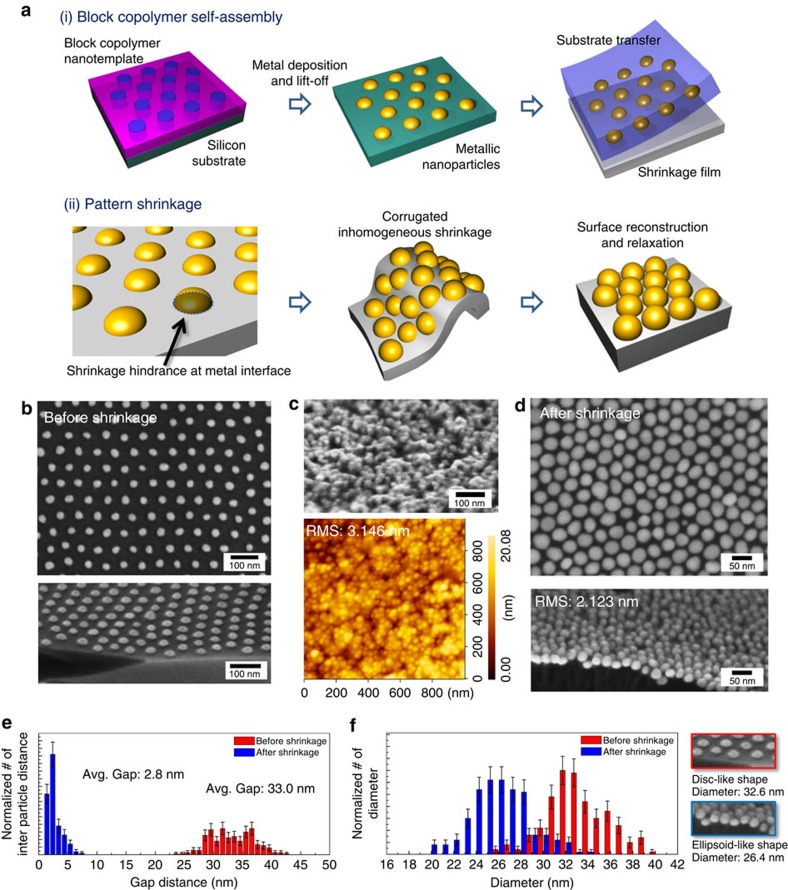
Formation of nanoparticle ensemble visible metamaterials. (**a**) Schematic for metal nanoparticle ensemble preparation by (i) BCP self-assembly and substrate transfer and (ii) pattern shrinkage. (**b**) SEM images of hexagonal Au nanoparticle arrays as-prepared from BCP self-assembly (upside: plane view, downside: 60° tilted view). (**c**) SEM and AFM images of intermediate corrugated nanopattern structure observed during lateral pattern shrinkage. (**d**) SEM images of Au nanoparticle ensembles after complete pattern shrinkage (upside: plane view, downside: 60° tilted view). (**e**,**f**) Distribution of interparticle distance (**e**) and particle diameter before (red) and after (blue) shrinkage (**f**). (The statistical errors in **e**,**f** are presented based on Poisson distribution.)

BCP self-assembly is well suited to realize this concept^33^,^34^,^35^,^36^,^37^,^38^. BCP generates well-ordered periodic nanostructures based on the microphase separation of immiscible polymer blocks. The intrinsic geometric shapes accessible by BCP self-assembly include diverse and complex configurations, from hexagonal cylinder arrays and parallel line arrays to double gyroids and other three-dimensional structures. Nonetheless, the topology is strongly correlated with the volume fractions of constitutive polymer blocks, governed by thermodynamics, and it is a huge challenge to vary the volume fractions by large amount while maintaining the desired topology. Accordingly, the achievable optical properties are also constrained. However, with the help of a controlled (an)isotropic shrinkage process, BCP can overcome the thermodynamic restrictions and provide an excellent platform for index control^33^,^34^. Figure 1 presents the formation of an Au nanoparticle ensemble with sub-10 nm interparticle distance, assisted by the shrinkage film. As shown in Fig. 1a, a pattern transfer method is utilized because direct BCP nanopatterning on the shrinkage film surface is impossible because of the low thermal/chemical stability of the shrinkage film^39^,^40^. First, a 85-nm-thick cylindrical polystyrene-*block*-poly(methyl methacrylate) (PS-*b*-PMMA) was spin-coated on a hard neutral substrate and thermally annealed to self-assemble into vertical hexagonal cylinder nanodomains^41^. An Au nanoparticle array with the height of ∼12 nm was generated by thermal evaporation and a lift-off process. The PMMA film layer was spin-casted upon the Au nanopattern as a transfer mediator. Substrate transfer on the shrinkage film was performed as described elsewhere^39^. No apparent lattice distortion of the nanoparticle array was observed during the substrate transfer (Supplementary Fig. 1).

Subsequent heat treatment at ∼180 °C triggered lateral film shrinkage down to ∼40% from its original dimension (Supplementary Movie 1 and snapshots in Supplementary Fig. 2). As shown in Fig. 1d and Supplementary Fig. 1, the interparticle distance (*g*) was dramatically reduced while the hexagonal ordering was maintained. The initial and final interparticle separations (s.d.'s; Fig. 1b,d) were measured to be 33.0 (3.8) and 2.8 (1.0) nm, respectively (Fig. 1e). The average particle diameter (*d*) was also modified from 32.6 (6.7) to 26.4 (2.6) nm (Fig. 1f). The resultant large *d/g* ratio (∼10) after pattern shrinkage is not allowed in a typical cylindrical self-assembly, where the self-assembled morphology evolves with the relative volume fraction of two polymer blocks^33^. The shrinkage ratio is controllable over a broad range by the processing parameters of the shrinkage film, such as the initial stretching ratio^42^.

The detailed mechanism underlying the uniform nanoscale pattern shrinkage was investigated by atomic force microscopy and scanning electron microscopy (SEM) analyses. The pattern shrinkage was found to proceed via two stages (Fig. 1a). Initially, Au nanoparticles have good interfacial adhesion to the shrinkage film, as confirmed by the tight contact without pattern distortion during the substrate transfer. Owing to this strong adhesion, the nanoscale surface regions of the shrinkage film attached to Au nanoparticles would be temporarily immobilized while the uncovered region as well as the bulk starts to shrink. We suspect that the resultant accumulation of inhomogeneous stress is the cause of the highly corrugated intermediate structure (Fig. 1c). The corrugation relaxes by a further heating process, during which Au nanoparticles might experience surface reconstruction owing to the enhanced mobility of Au at the elevated temperature (Supplementary Fig. 3)^43^. The size and shape change of the nanoparticles supports this surface reconstruction model to form the low-energy state of near-spherical particles (Fig. 1f and Supplementary Fig. 4).

### Optical properties of self-assembled visible metamaterials

**Figure 2:**
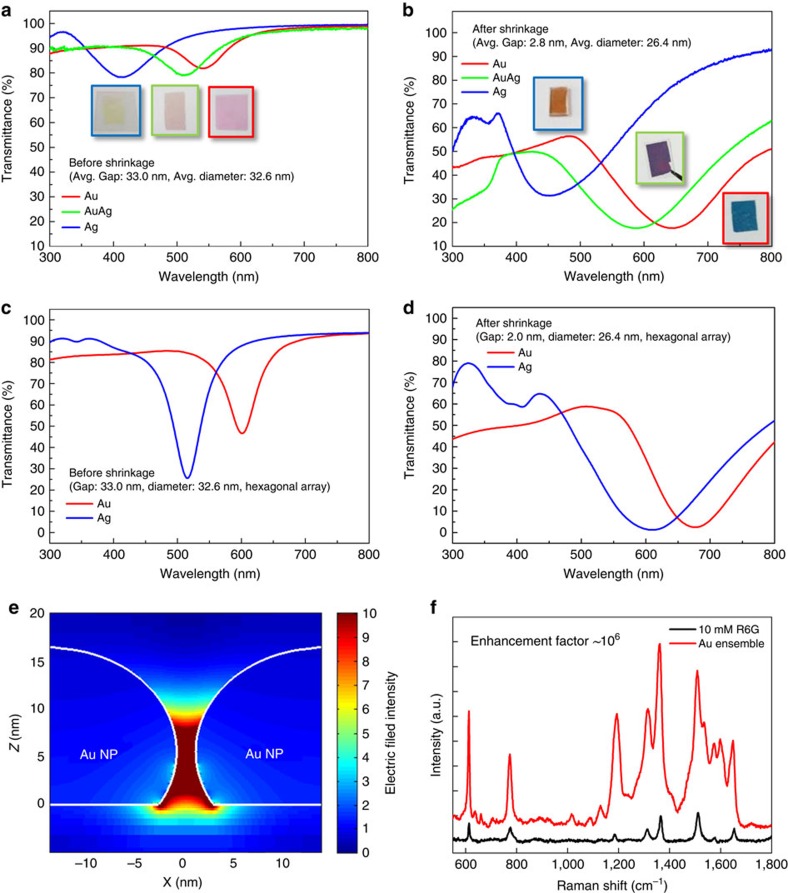
Optical properties of metallic nanoparticle ensemble before and after shrinkage. (**a**,**b**) Ultraviolet–vis spectroscopy and photographs of Au, Ag and Au–Ag alloy nanoparticle ensemble before (**a**) and after shrinkage (**b**). (**c**,**d**) Corresponding FDTD transmittance simulations of Au, Ag nanoparticle ensembles. (**e**) Simulated intensity profile of electric field between neighbouring Au nanoparticles. (**f**) Surface-enhanced Raman spectroscopy of Au nanoparticle ensemble for R6G.

The optical properties of Au, Ag and Au–Ag alloy (Au 0.63 at%-Ag 0.37 at%) particle ensembles were characterized with a spectrometer (Fig. 2). The colour change was obvious during shrinkage (Fig. 2a,b). Metallic nanoparticles before shrinkage displayed red (Au), yellow (Ag) and pink (Au–Ag alloy) colours, as determined by the typical localized surface plasmon resonances (Au: ∼550 nm, Ag: ∼400 nm, Au–Ag alloy: ∼500 nm). The colours shifted to blue (Au), orange (Ag) and purple (Au–Ag alloy) after shrinkage, and broad extinction at ∼650 nm (Au), ∼450 nm (Ag) and ∼590 nm (Au–Ag alloy) was observed. The red-shift and broadening of resonances are attributed to the weakened restoring force on oscillating electrons in nanoparticles caused by the strong near-field coupling of neighbouring particles in close proximity^44^.

The observed optical properties of nanoparticle ensembles match well with finite-difference time-domain (FDTD) simulations. The resonance dips obtained from a FDTD simulation clearly red-shift after the interparticle distance decreases (Fig. 2c,d). The relatively large difference of the numerical and experimental results for the Ag ensemble is because of the surface oxidation of Ag, which decreases the diameter of the metallic cores, increasing the intercore distances as a result, and changes the average permittivity of the gap-filling dielectrics. In addition, broader widths of the extinction bands (Fig. 2b) compared with the simulation (Fig. 2d) are associated with the distribution of the interparticle distance (Fig. 1e and Supplementary Fig. 5) as well as particle size inhomogeneity (Fig. 1f). Meanwhile, the strong near-field coupling between nanoparticles was confirmed by the field intensity profile (Fig. 2e). This capacitive coupling and field enhancement also could be verified by surface-enhanced Raman spectroscopy (SERS). SERS for rhodamine 6G on an Au nanoparticle ensemble showed a high enhancement factor of ∼10^6^ (Fig. 2f and Supplementary Fig. 6).

**Figure 3:**
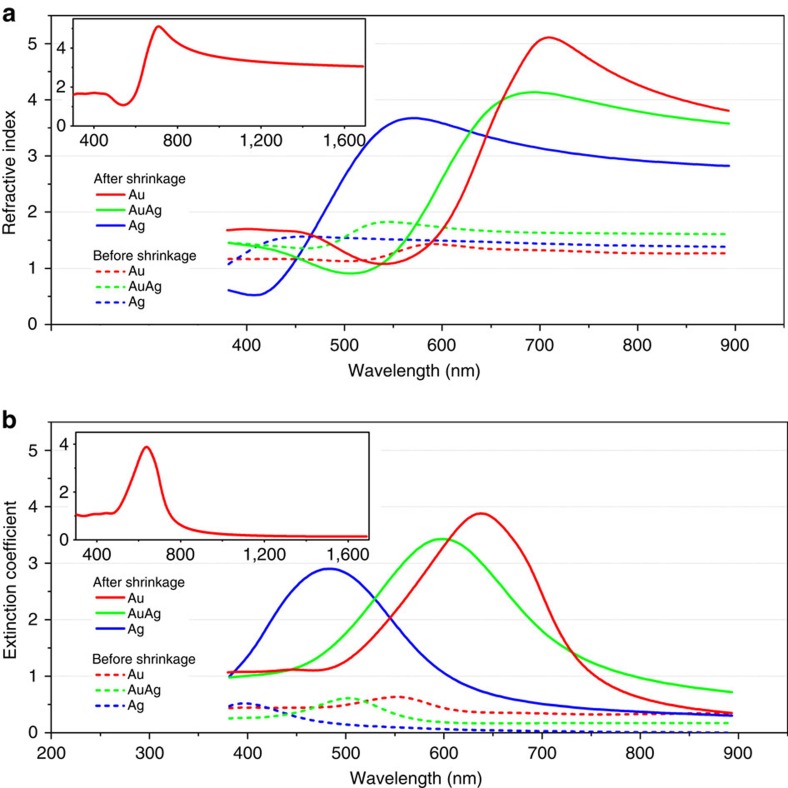
Refractive index of metallic nanoparticle ensemble. Ellipsometry measurements of (**a**) refractive indices and (**b**) extinction coefficients of Au, Ag and Au–Ag alloy nanoparticle ensembles before (dashed line) and after (solid line) pattern shrinkage.

For effective refractive index measurements, ellipsometry was performed (Fig. 3 and Supplementary Fig. 7). Before shrinkage, the coupling between nanoparticles is insignificant because of large interparticle distances. Accordingly, a low *n*_eff_ was measured (Fig. 3 (dashed lines) and Table 1). By contrast, *n*_eff_ increased substantially after shrinkage, up to more than threefold (Fig. 3 (solid lines), Supplementary Fig. 7 and Table 1) to a peak value of 5.10 at a wavelength of 709 nm for Au. The *n*_eff_ for the Au metamaterial is above 3.0 over a wavelength range from 638 to 1,700 nm (upper wavelength limit of our ellipsometer; Fig. 3 inset). The imaginary part of the index, which is related to optical losses, exhibits a high peak value of 3.9 at 640 nm wavelength but becomes less than 0.5 at 840 nm and continues to decrease at longer wavelengths. (The high values of the imaginary part near the resonance wavelength are associated with the high values of the real part over broad bandwidth in the long wavelength regime via the Kramers–Kronig relationship.) Such a high *n*_eff_ with low loss and broad bandwidth in the near-infrared and the red part of the visible range is beyond the values achievable by previously reported artificial materials or natural dielectrics^45^ except several semiconductor materials such as Si and GaAs. Recently reported topological insulators possess even higher index approaching 5.5 in this wavelength range^46^; however, the extinction coefficient is also quite high at near 2 or higher. Notably, this non-resonant enhancement is in contrast to other metamaterials relying on structural resonances of individual metallic inclusions, which is inherently narrow-band and sensitive to the optical loss of metals^47^. The ellipsometry results were based on a commercial ellipsometry algorithm (CompleteEASE), which assumes *μ*_eff_=1. The validity of this assumption for our samples was confirmed by FDTD simulations (Supplementary Fig. 8). Numerically, both *ɛ*_eff_ and *μ*_eff_ can be retrieved from the complex transmission and reflection coefficients obtained by FDTD simulations^48^. The results showed highly increased *ɛ*_eff_ and near-unity *μ*_eff_, as predicted by the theoretical model (Supplementary Fig. 8). The refractive index was expected to continuously increase until an interparticle distance of 1 nm (Supplementary Fig. 9). Moreover, it was confirmed that the incident-angle-dependent optical response of this metamaterial is consistent with that of the uniform uniaxial crystal thin film (Supplementary Fig. 10 and Supplementary Discussion).

### High controllability of self-assembled visible metamaterials

**Figure 4:**
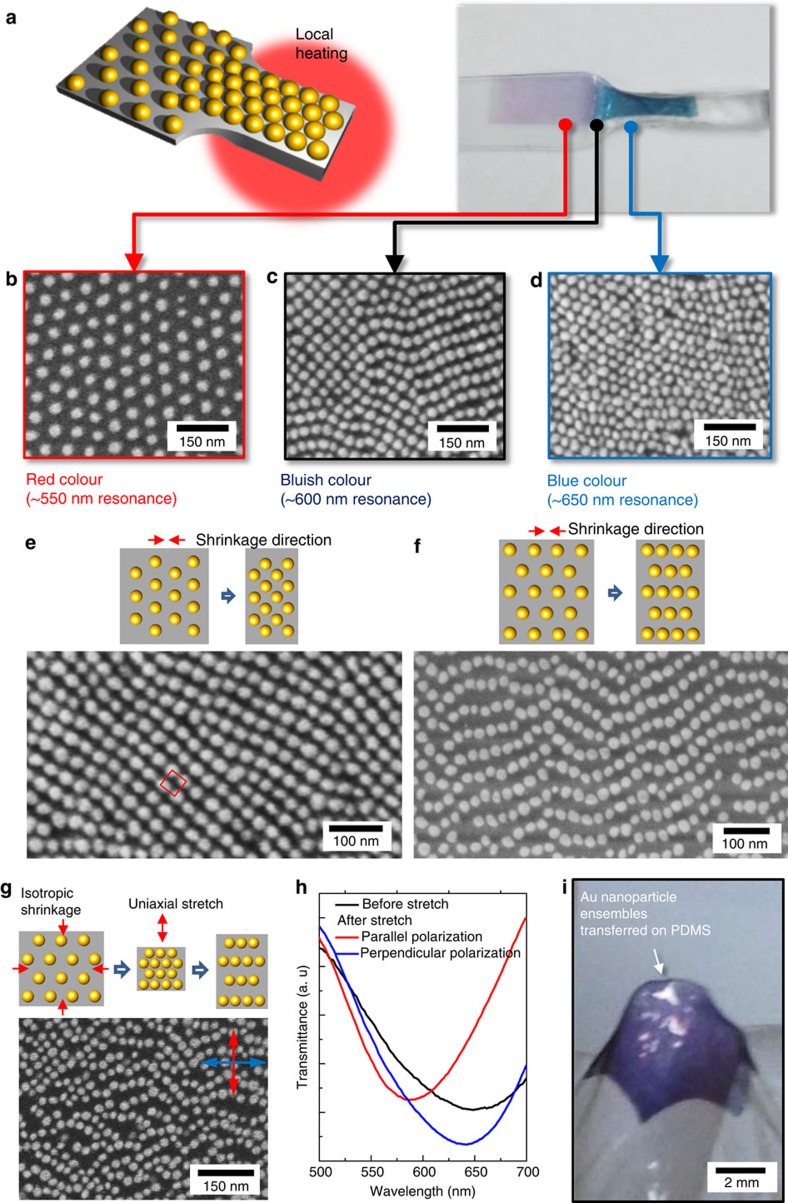
Tunable refractive index metamaterials via symmetry modification. (**a**) Schematic and photograph of nanoparticle ensemble with gradually varying interparticle distance by inhomogeneous thermal shrinkage. (**b**–**d**) Corresponding SEM images of unshrunken region (**b**), intermediate region (**c**) and fully shrunken region (**d**). (**e**,**f**) Nanoparticle ensembles with fourfold symmetry (square array; **e**) and twofold symmetry obtained from anisotropic shrinkage of hexagonal array in different directions (**f**). (**g**,**h**) SEM image anisotropic nanoparticle ensemble obtained from unidirectional stretching (**g**) and its birefringent UV-vis spectroscopy in response to polarized light (**h**). (**i**) Photograph of Au nanoparticle ensemble transferred on a conventional PDMS film surface.

Motivated by the strong influence of an ensemble configuration on the optical properties of metamaterials, effective modification of the intrinsic hexagonal symmetry of the nanoparticle array and the corresponding optical properties were explored. First, the spatially invariant periodicity of the BCP pattern indicates that the resulting optical properties also have translational invariance. Although the BCP nanopattern shows a multigrain nature due to the entropy-induced grain freedom, it has sixfold rotational symmetry within each grain. Consequently, the effective optical properties are isotropic for the in-plane fields and do not depend on the orientation of the grain in the long wavelength limit^49^. Thus, the interparticle distance (*g*) and the particle sizes (*d*) are the only significant parameters, and the spatially uniform *n*_eff_ could be obtained before and after shrinkage. Interestingly, the refractive index gradient is also easily achievable by spatial control of *g*. We performed localized heating of the shrinkage film and induced a spatial gradient of the shrinkage ratio (Fig. 4a–d). This leads to a gradually varying *n*_eff_ profile, decreasing from the dense nanoparticle region to the sparse region. Many applications can also benefit from a controlled spatial gradient of *n*_eff_, among which a graded-index lens is a prime example^50^,^51^,^52^,^53^.

Substrate engineering using a shrinkage film can also modify the rotational symmetry of BCP patterns in a well-controlled manner. The hexagonal cylindrical phase used in this work shows sixfold rotational symmetry (C_6_) in the equilibrium form. Anisotropic shrinkage was induced by holding the two opposite sides of a shrinkage film with a set of rigid bars and allowing the distance between the bars to shrink. This controlled anisotropic shrinkage realized fourfold (C_4_) or twofold (C_2_) rotational symmetry (Fig. 4e,f). The fourfold symmetry (square array) is unavailable with conventional diblock copolymer self-assembly. For twofold symmetry, anisotropic optical coupling occurs. Numerical results exhibit maximum birefringence of 4.13 in refractive index units on resonance (575 nm wavelength) for silver particles with dimensions described in Methods with an interparticle distance of 1 nm in the *x* direction, which is much higher than previously reported high birefringent materials^54^ (Supplementary Fig. 11).

When a ‘multigrain' BCP pattern is anisotropically shrunken, a variety of configurations with different symmetries are expected in the same film (Supplementary Fig. 12). A better uniformity could be obtained if the sample was isotropically shrunken first and then stretched unidirectionally (Fig. 4g). The optical property of this anisotropic sample was measured by polarizer-equipped ultraviolet–visible (vis) spectroscopy (Fig. 4h). For the incident light polarized parallel to the shrinkage direction, the optical resonance was similar (resonance wavelength at ∼640 nm) to that of the isotropically shrunken sample (Fig. 4h, blue line). By contrast, for the orthogonally polarized light, the resonance was observed at ∼585 nm (Fig. 4h, red line). Notably, a similar level of anisotropy cannot be observed when the original hexagonal nanoparticle arrays are anisotropically stretched because near-field coupling does not occur in either direction (Supplementary Fig. 13).

For practical applications, the prepared visible metamaterials with controllable *n*_eff_ can be transferred to various substrates (Fig. 4i and Supplementary Fig. 14). The nanoparticle arrays can also be laterally patterned into desired shapes such as optical cavities, couplers and waveguides (Supplementary Fig. 15)^19^,^35^. Since the effective wavelength is greatly reduced in the high refractive index medium, this approach may serve as a bridge between conventional optics and plasmonics or even provide a lower-loss alternative to plasmonics in the quest for miniature optical components.

## Discussion

Precise spatial density control of BCP nanopatterns by pattern shrinkage realized strong near-field capacitive coupling while suppressing the diamagnetism through metallic inclusion of smaller dimension than the skin depth, which is essential for the effective enhancement of *n*_eff_. This enhancement was achieved in a broad wavelength range including the visible spectrum due to utilization of the non-resonant nature. In addition, versatile controllability of pattern shrinkage afforded wide tunability of *n*_eff_, including spatially graded or anisotropic refractive index metamaterials.

This freedom of spatial and directional *n*_eff_ design may greatly strengthen the capability to manipulate visible light at surface-patterned structures and thereby have an impact on diverse areas including bioimaging, lithography, communications and transformation optics, where the propagation of light is highly important. Furthermore, symmetry and period modification by nanoscale pattern shrinkage can be implemented for other nanopatterning methods and self-assembling systems for various types of visible metamaterials^55^,^56^.

## Methods

### Preparation of metallic nanoparticle array by BCP lithography

For the vertical alignment of cylinder nanodomains in PS-*b*-PMMA BCP nanotemplates, the surface of a Si substrate was chemically modified by a PS-*r*-PMMA random copolymer brush (PS ratio: 0.62, 8 kg mol^−1^), after ultraviolet ozone treatment radiation or piranha solution immersion for 30 min. A cylindrical PS-*b*-PMMA (140 kg·mol^−1^-*b*-65 kg·mol^−1^) thin film with ∼85 nm thickness was then spin-coated and thermally annealed over 4 h to obtain the self-assembled morphology with surface vertical cylinder nanodomains. The PMMA nanodomains were selectively removed by diluted acetic acid immersion and oxygen reactive ion etching. Acetic acid immersion was performed for 10 min and was thoroughly washed with deionized (DI) water, and oxygen reactive ion etching was performed at 50 W radio frequency (RF) power and 0.07 torr. To form the metallic nanoparticle array, metal deposition was performed on the PS nanoporous template remaining on the Si surface by an E-beam evaporator. A roughly 12-nm-thick metal (Au, Ag) layer was deposited. The lift-off process was fulfilled by thorough sonication in toluene to leave a metal nanopattern replicating the self-assembled morphology of the original BCP nanotemplate. For the Au–Ag alloy, the sequential deposition of Au and Ag with 5–7 nm thickness was performed and thermally annealed at 600 °C for 3 h.

### Nanopattern shrinkage by substrate transfer and thermal treatment

Period control of the metallic nanopatterns was performed with the assistance of the shrinkage film. To form the metallic nanoparticle array on the shrinkage film, a transfer method utilizing the PMMA thin film was utilized. A PMMA thin film (495PMMA A8, MicroChem, USA) with 500 nm thickness was spin-coated on the metallic nanoparticle array formed on the Si substrate, and thermally annealed at 100 °C for 30 min. The sample was immersed in 3 M KOH aqueous solution for several minutes to hydrolyse the PMMA. Subsequent immersion of the sample in a water bath fully separated the PMMA film embedded with the nanoparticle array from the Si substrate. This process made the floating PMMA film containing the nanoparticle array at the water surface. It was carefully transferred to the shrinkage film (Polyshrink, Lucky Squirrel, USA). After complete drying, the PMMA layer was chemically removed by an acetic acid solution and thoroughly washed with DI water. Acetic acid selectively removed the PMMA without damage of the substrate. Post-heat treatment was performed at 180 °C for 2–3 min. For the anisotropic shrinkage, the sample was heated while fixing one side of the sample by a home-made stiff hard bar.

### Transmission measurement

For the measurement of optical properties, commercially available ultraviolet–vis spectroscopy (UV-2600, SHIMADZU) was used. A specular transmission mode was utilized after normalization for the shrinkage film. For the polarization-dependent measurement, film polarizer with an operating wavelength range of 400–700 nm (LPVISE200-A, THOR LABS) is equipped at the front of the spectroscopy source.

### Ellipsometer measurement

An ellipsometer (alpha-SE ellipsometer or M-2000, J.A. Wollam) was utilized to obtain the refractive index of the metallic nanoparticle array in the visible/infrared region. A 70° tilting angle and ‘long' measuring mode were selected. For exact measurements, the refractive index of the shrinkage film without a nanoparticle array was measured by the Cauchy model before and after shrinkage. There was no significant difference owing to their similar density. The refractive index and the thickness of the nanoparticle array on the shrinkage film were measured by the B-spline model, considering their absorption properties. The spot size of the light source is ∼9 mm^2^.

### SERS

A High-Resolution Dispersive Raman Microscope (ARAMIS, Horiba Jobin Yvon) was used for SERS measurement of R6G. The 633 nm (He-Na laser) light source was applied to the sample in order to match the resonance wavelength of the close-packed Au nanoparticle ensemble. A × 10 objective and acquisition time of 10 s were used for all measurements. As a reference, the Raman spectrum was obtained from 10 mM R6G in water. The number of molecules probed within a focal laser spot was calculated by focal diameter of 1 μm and focal depth of 20 μm. For SERS measurement, a 100 nM solution of R6G in ethanol was dropped on a 1 cm × 1 cm area of the Au nanoparticle ensemble and dried. To evaluate the enhancement factor, the intensities at 1,310, 1,360, 1,510 and 1,650 cm^−1^ were quantified.

### Transfer of nanoparticle ensemble to various substrates

For a hard substrate such as silicon, the nanoparticle ensemble was heated above the glass transition temperature of the shrinkage film under pressure, facing the nanostructure on the shrinkage film towards the target substrate, to promote good adhesion. For soft substrates such as PDMS, polymeric substrate materials were deposited and cured, after surface treatment of metal particles by self-assembled monolayer deposition. Subsequently, the polymeric shrinkage film was chemically removed.

### FDTD simulation

Lumerical FDTD Solutions 8.15 was used for the calculation. The size of uniform mesh cells was set at 0.1 nm. A plane wave propagating along the *z* axis was used as the excitation source, with 300–1,200 nm wavelength range. While the boundary condition for the *x*- or *y* directions was symmetric or antisymmetric, depending on the incident polarization, accounting for the periodicity of the structure, that of the z direction was a perfect matched layer consisting of 128 layers to minimize unphysical reflection from the simulation boundary. Material properties of gold and silver are adopted from Palik's optical constants handbook^41^. The geometric parameters of the structure before and after shrinkage were obtained from SEM and transmission electron microscopy results. Before shrinkage, the shapes of metal particles were approximated as oblate hemispheroids with 32.6 nm of the major axis and 12.8 nm of the minor semi-axis (height). After shrinkage, on the other hand, the shapes were assumed to have changed to truncated oblate spheroids with a 26.4 nm major axis, a 10.82 nm minor semi-axis and 16.28 nm height. The lattice constant of the structure before and after shrinkage was set to be 65.6 and 28.4 nm with the hexagonal lattice unless specified otherwise.

### Data availability

The data that support the findings of this study are available from the corresponding author upon request.

## Additional information

**How to cite this article:** Kim, J. Y. *et al*. Highly tunable refractive index visible-light metasurface from block copolymer self-assembly. *Nat. Commun.* **7,** 12911 doi: 10.1038/ncomms12911 (2016).

## Supplementary Material

Supplementary Information Supplementary Figures 1-15 and Supplementary Discussion.

Supplementary Movie 1 Real-time visual change during the pattern shrinkage process.

## Figures and Tables

**Table 1:** Summary of measured refractive index of metamaterials.

	**Peak refractive index**
*Before shrinkage*	
Au	1.43 (583 nm)
Au–Ag alloy	1.83 (544 nm)
Ag	1.56 (460 nm)
	
*After shrinkage*	
Au	5.10 (709 nm)
Au–Ag alloy	4.16 (690 nm)
Ag	3.67 (567 nm)
