# Supplementary material for: Highly tunable refractive index visible-light metasurface from block copolymer self-assembly
Source: Nat Commun. 2016 Sep 29;7:12911. doi: 10.1038/ncomms12911 (PMC5056414; doi:10.1038/ncomms12911)
Supplement: Supplementary Information — Supplementary Figures 1-15 and Supplementary Discussion. [file ncomms12911-s1.pdf]

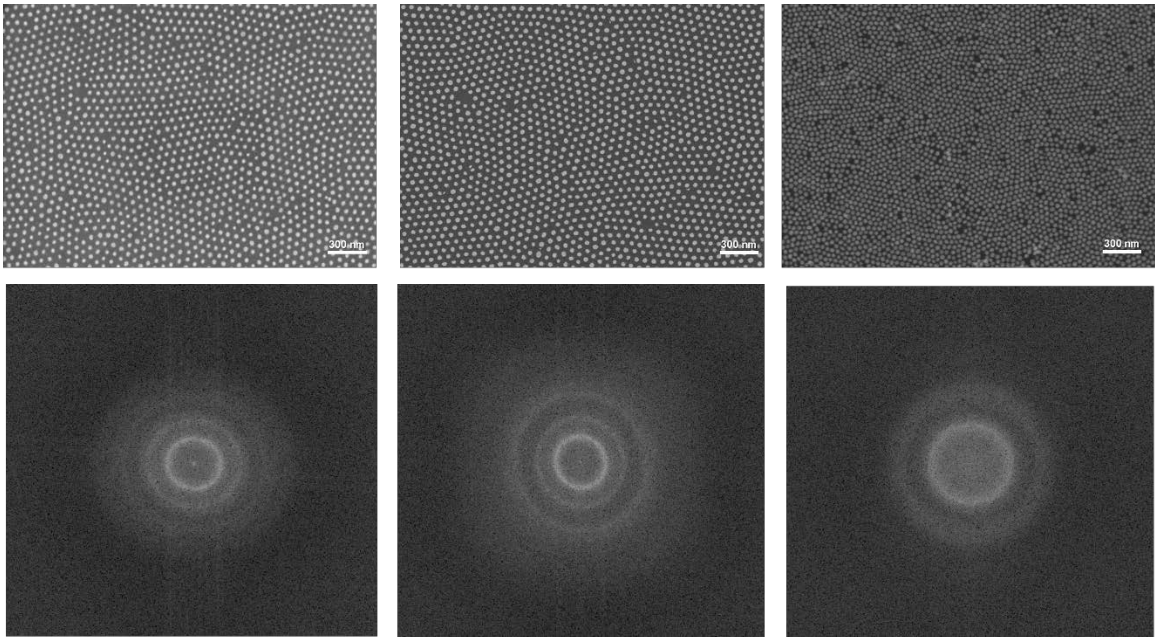

Supplementary Figure 1 | SEM images and corresponding Fourier Transformation of nanoparticle arrays before pattern transfer (left), after pattern transfer but before pattern shrinkage (middle), and after pattern shrinkage.

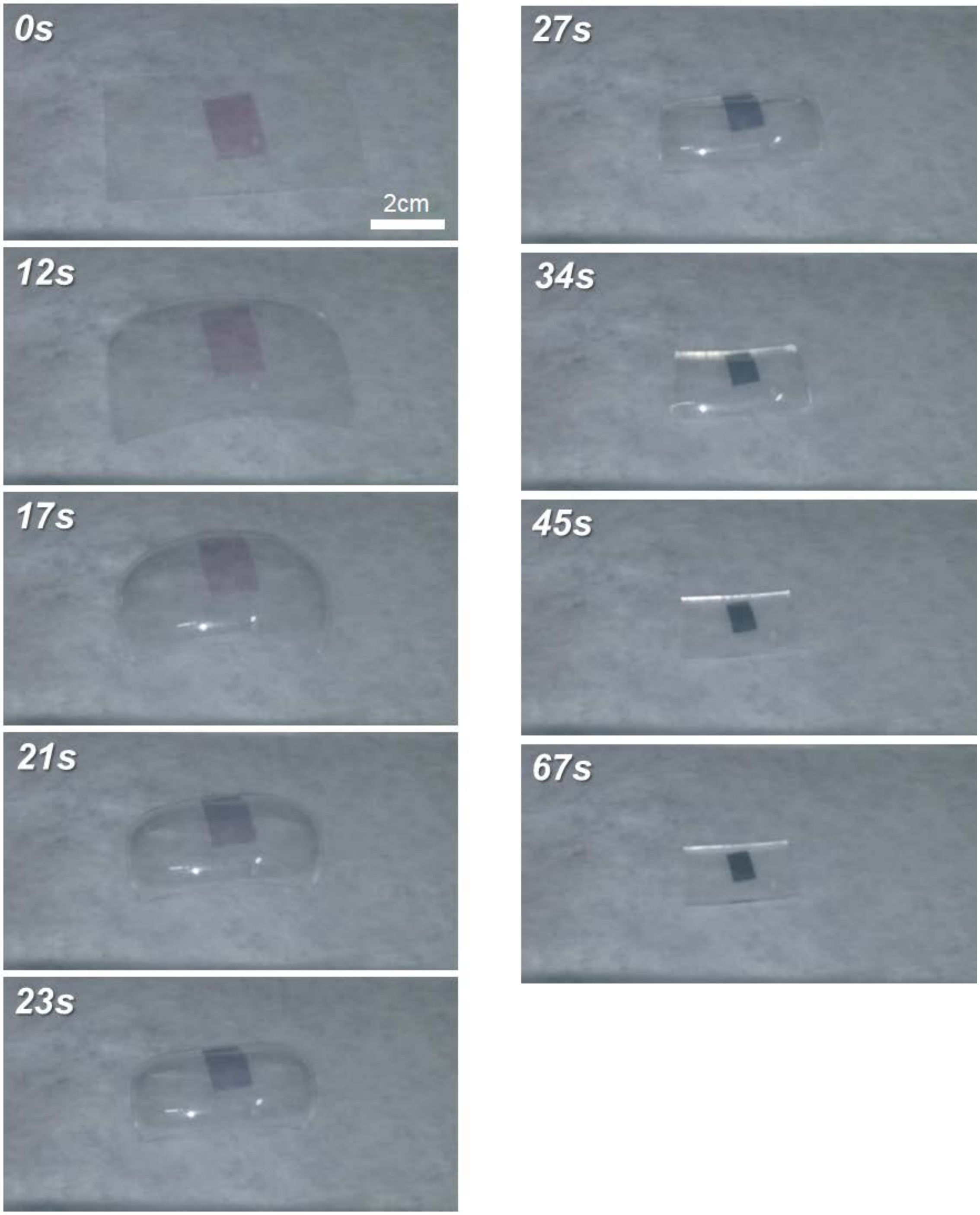

Supplementary Figure 2 | Snap shot images during pattern shrinkage process. Real time images were presented, presenting the color change of Au nanoparticle array from red to blue

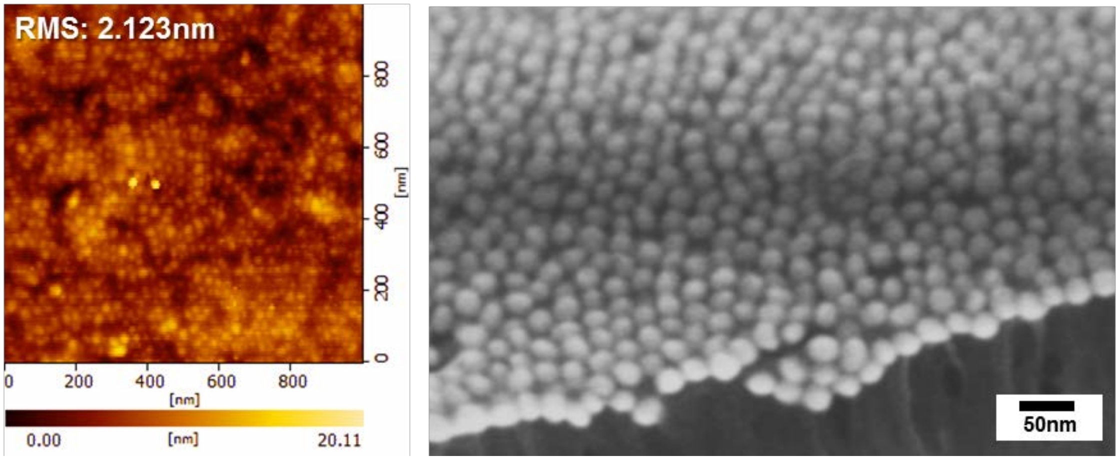

Supplementary Figure 3 | AFM & SEM images of flat structure with nanoparticle array after completion of shrinkage process

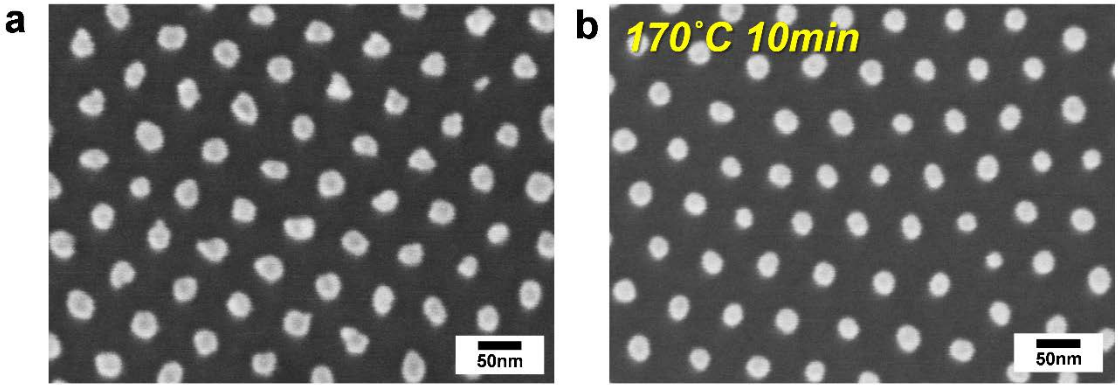

Supplementary Figure 4 | a, **b**, SEM images of Au nanoparticle arrays on silicon substrates before (**a**) & after (**b**) thermal annealing at 170 °C for 10 min.

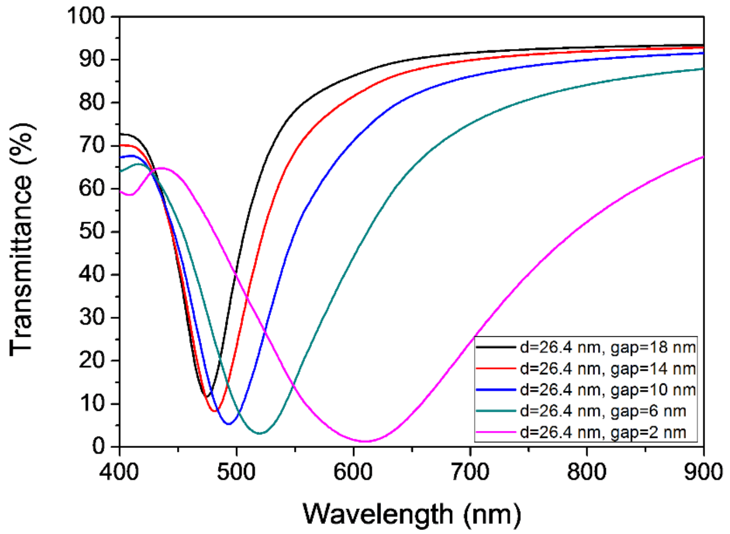

Supplementary Figure 5 | Simulated resonance frequency of nanoparticle ensembles as a function of inter-particle distance

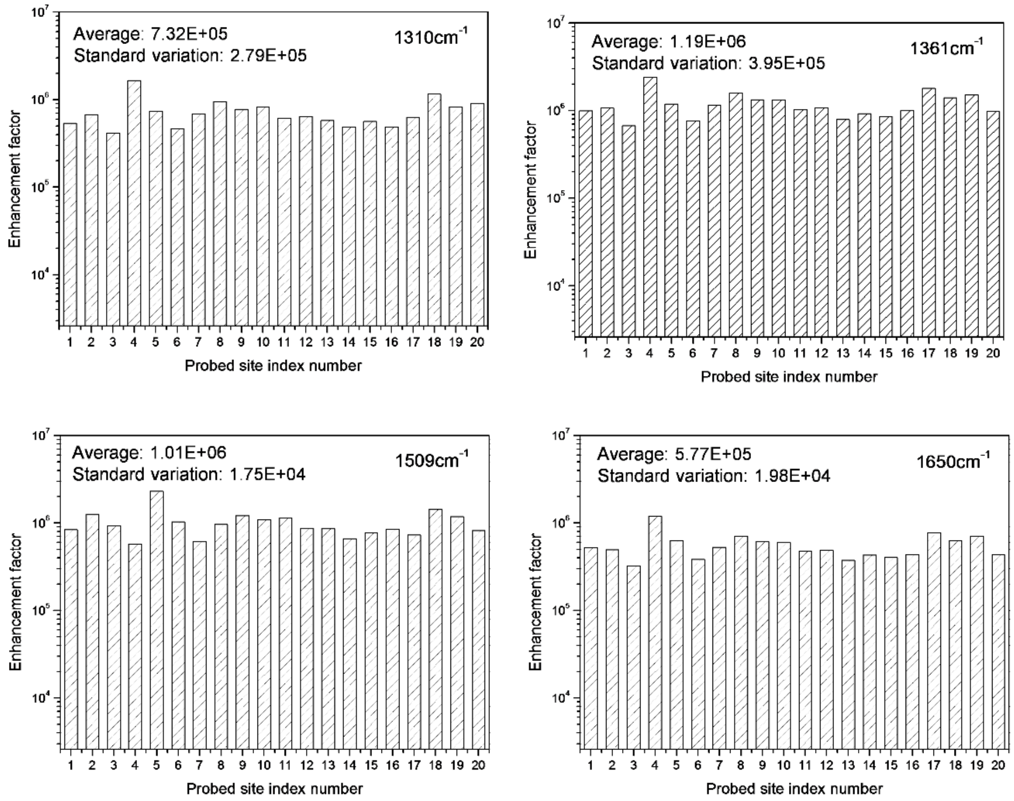

Supplementary Figure 6 | Uniform SERS of Au nanoparticle ensembles prepared from the pattern shrinkage process.

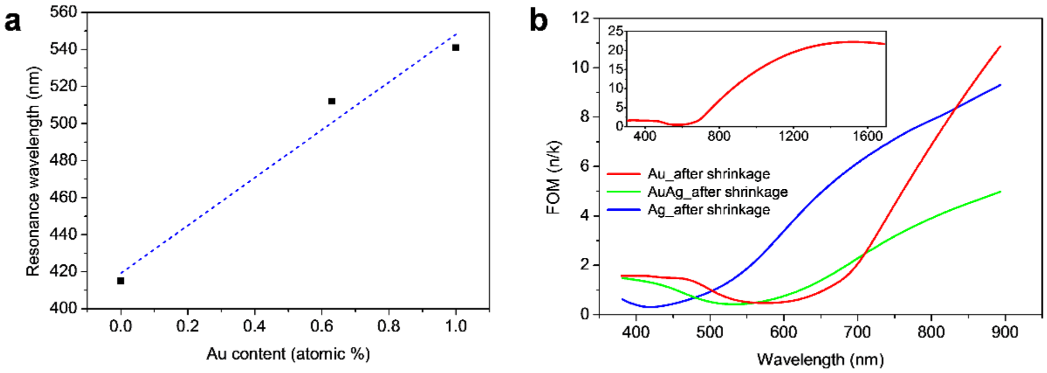

Supplementary Figure 7 | a, Resonance wavelength shift as a function of Au content. **b**, Figure of merit (FOM = refractive index / extinction coefficient) of each metamaterial.

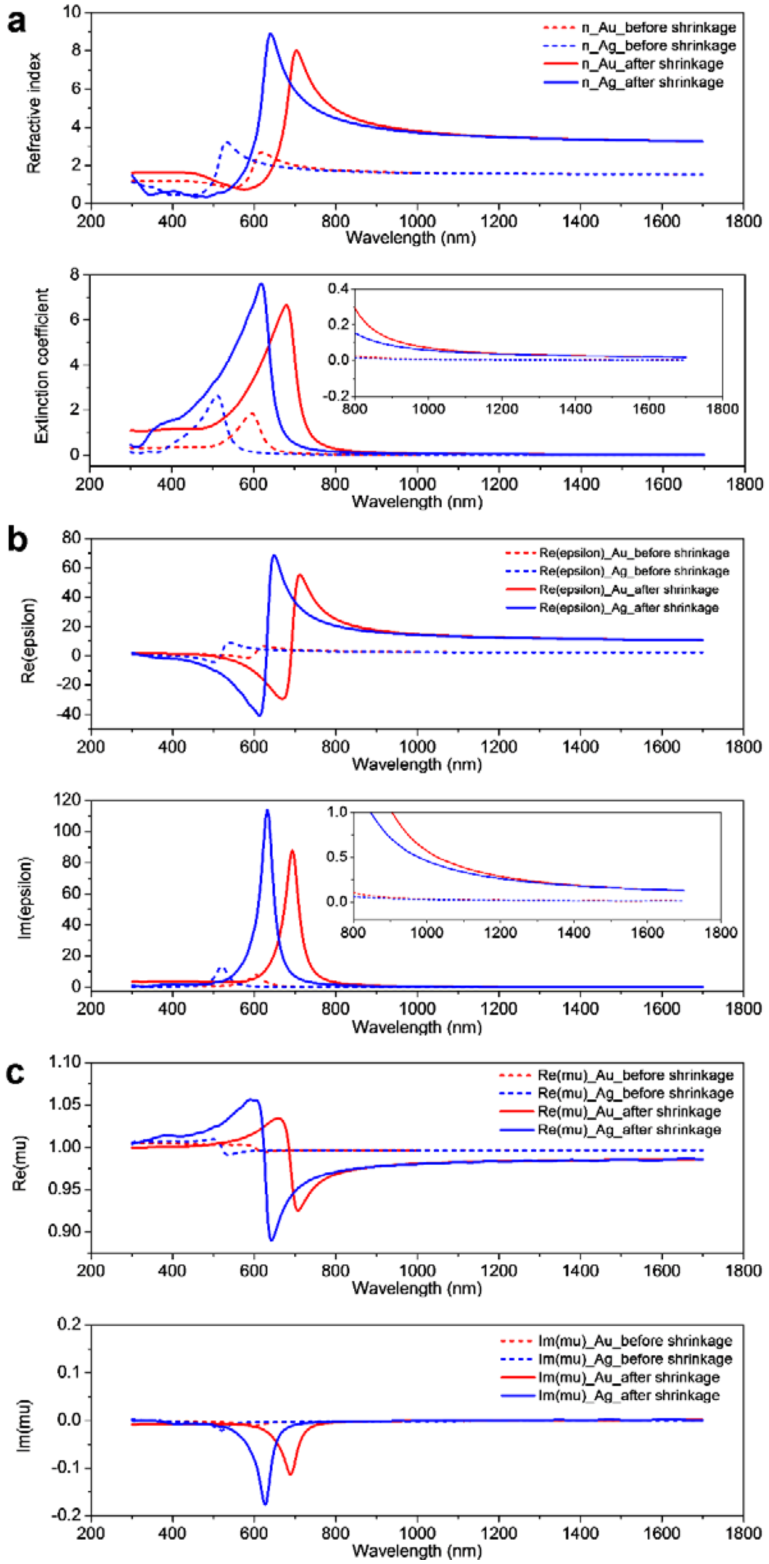

Supplementary Figure 8 | a-c, Complex refractive index (**a**), permittivity (**b**) and permeability (**c**) obtained from retrieval method of scattering parameters.

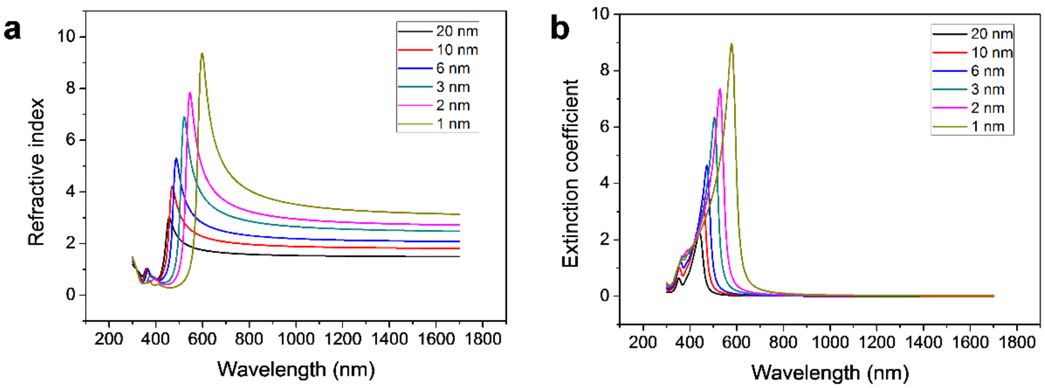

Supplementary Figure 9 | Simulated **a**, refractive index and **b**, extinction coefficient for various inter-particle distances. The particle shape is fixed as a truncated spheroid with 26.4 nm major axis, 10.82 nm minor semi-axis, and 16.28 nm height. The lattice is hexagonal with C_6 symmetry.

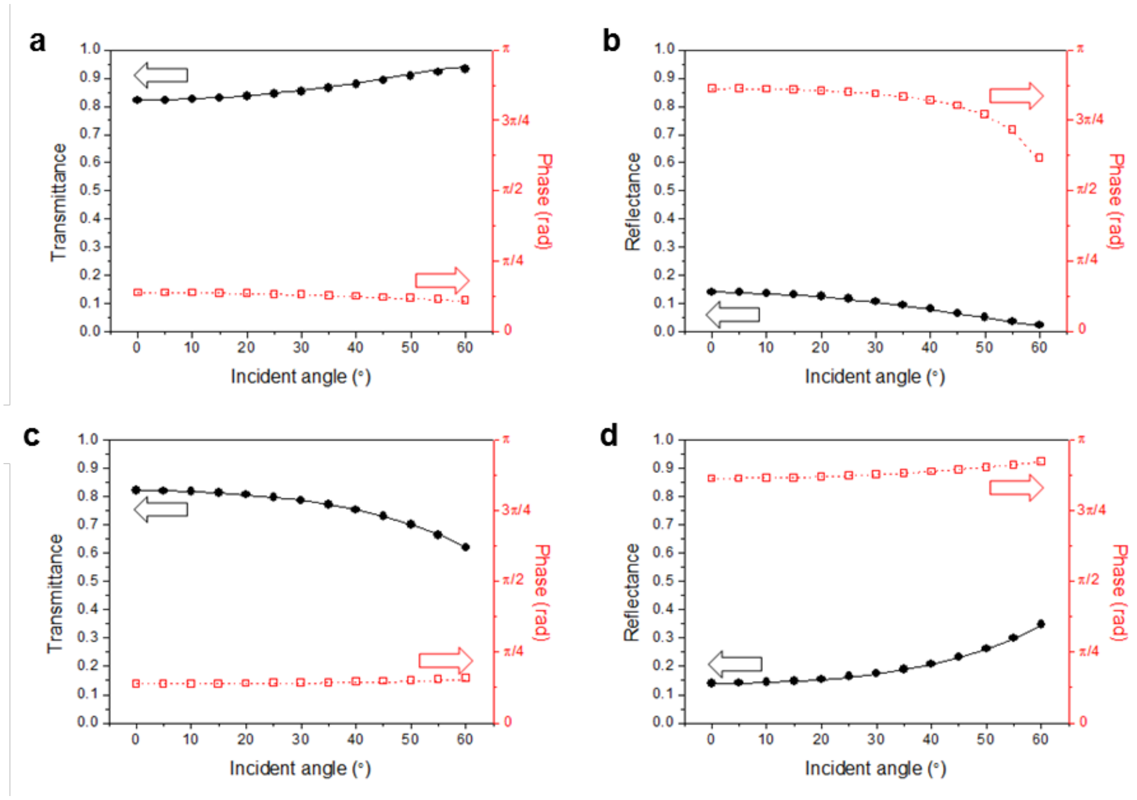

Supplementary Figure 10 | Angle dependence of **a**, transmission and **b**, reflection for p-polarized incident light of 1000 nm wavelength. Intensities are shown with block solid lines (uniform thin film) and black dots (metasurface) and the phases are plotted with red dotted lines (uniform thin film) and red open squares (metasurface). **c**, and **d**, are for s-polarized incident light.

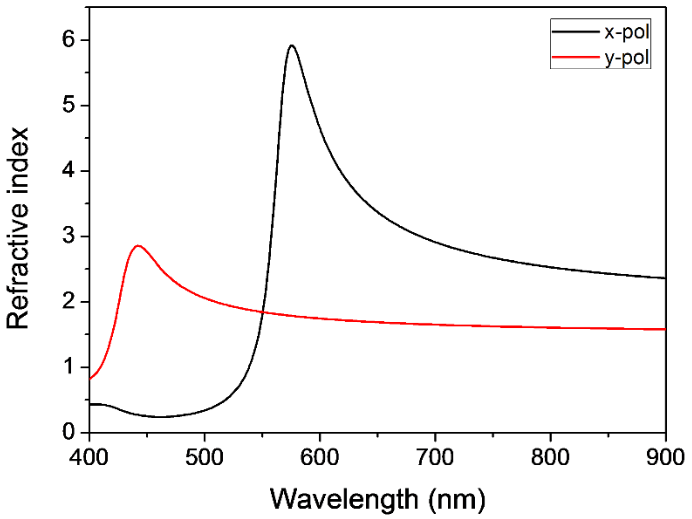

Supplementary Figure 11 | Polarization dependent refractive index from FDTD calculations of anisotropically coupled Ag nanoparticle ensemble with 1 nm inter-particle distance in x direction.

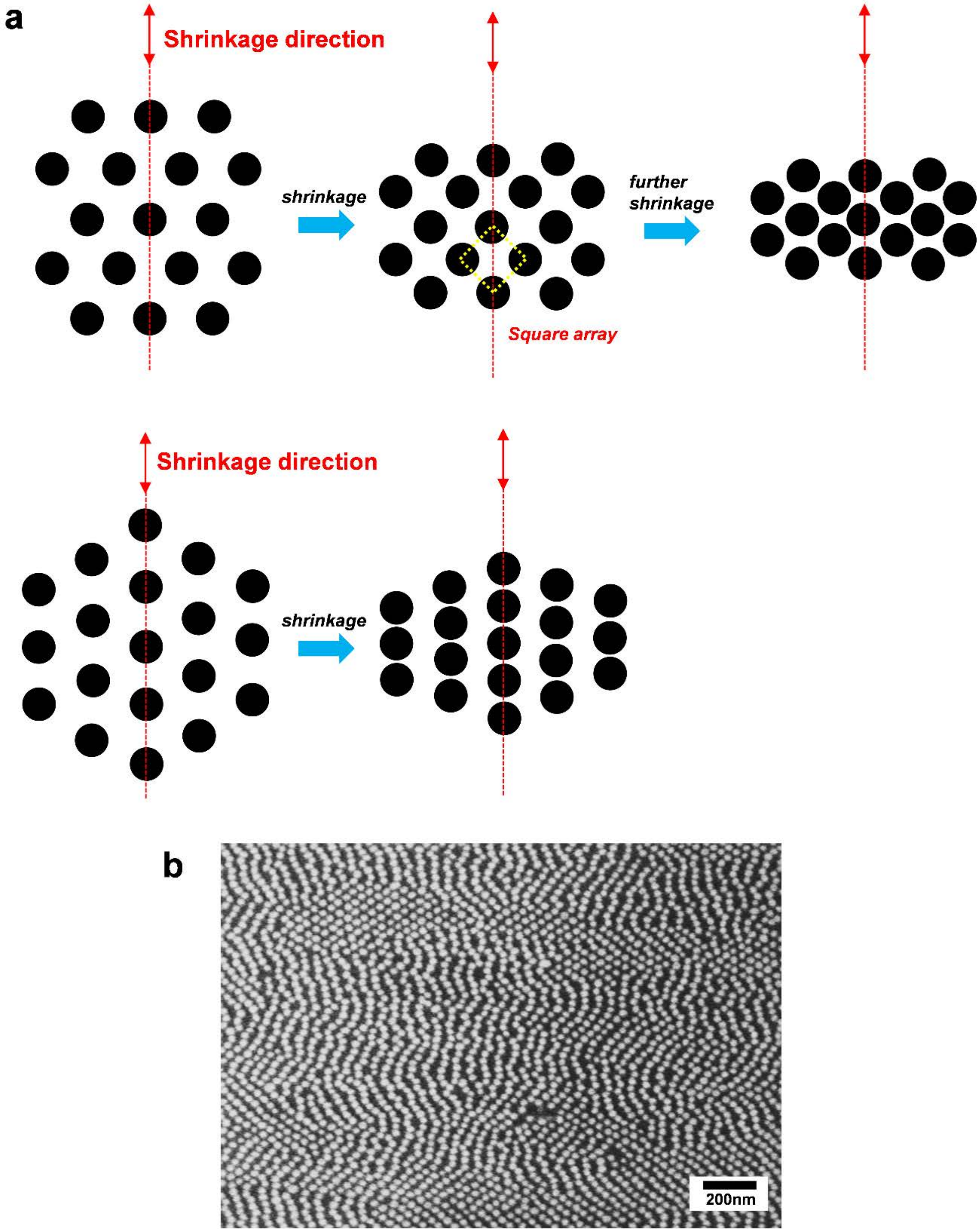

Supplementary Figure 12 | a, Morphological change of nanoparticle arrays with 6-fold symmetry depending on the shrinkage direction and grain orientation. **b**, Due to the dependency to shrinkage direction and randomness of BCP grain orientation, anisotropic shrinkage results in the coexistence of complex morphologies of nanoparticle arrays.

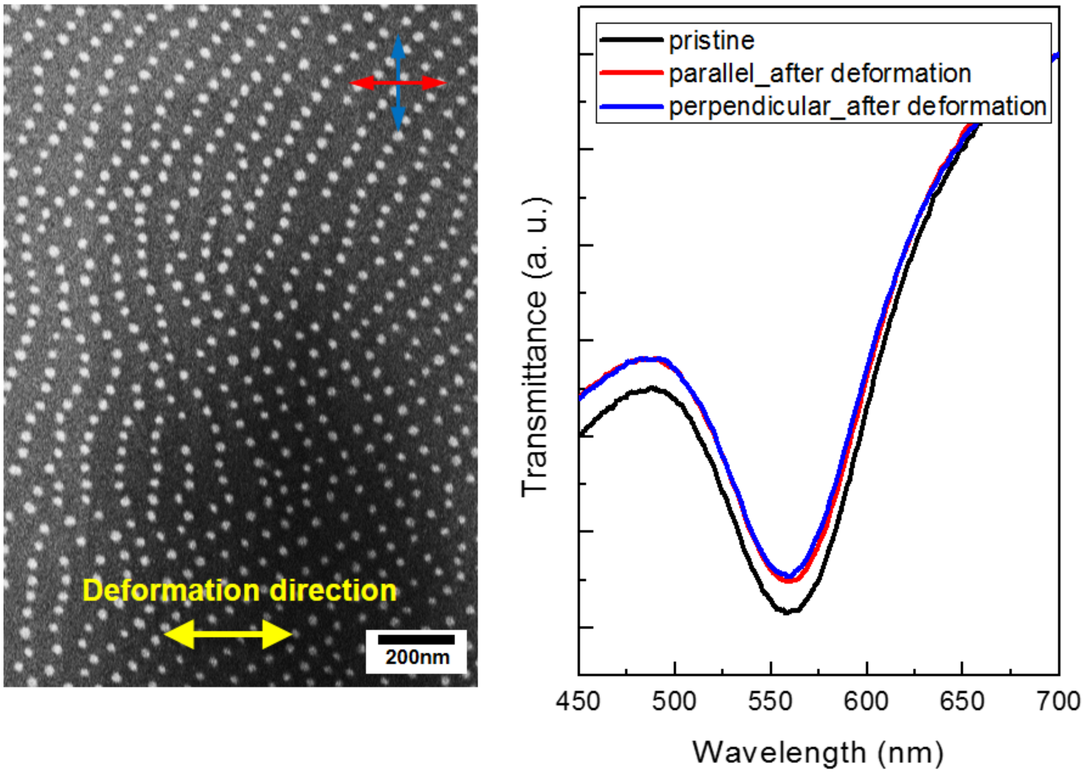

Supplementary Figure 13 | Control experimental for anisotropic elongation of nanoparticle array with sparse inter-particle distance, and its optical responses to polarized light.

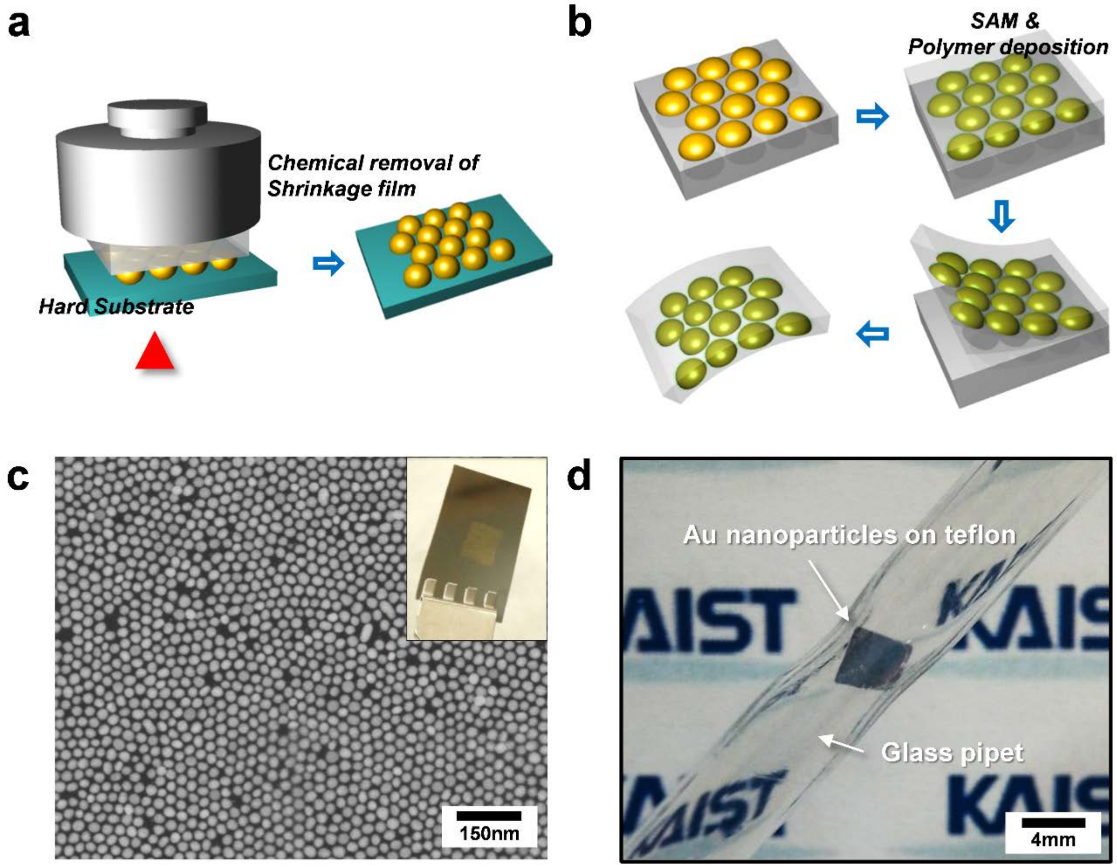

Supplementary Figure 14 | Substrate transfer of nanoparticle ensemble visible metamaterials onto hard or soft substrate. **a**, **b**, Schematic illustrations for the transfer onto hard (**a**) and soft substrate (**b**). **c**, **d**, SEM and photographic images after transfer onto silicon (**c**) and glass pipet surface (**d**).

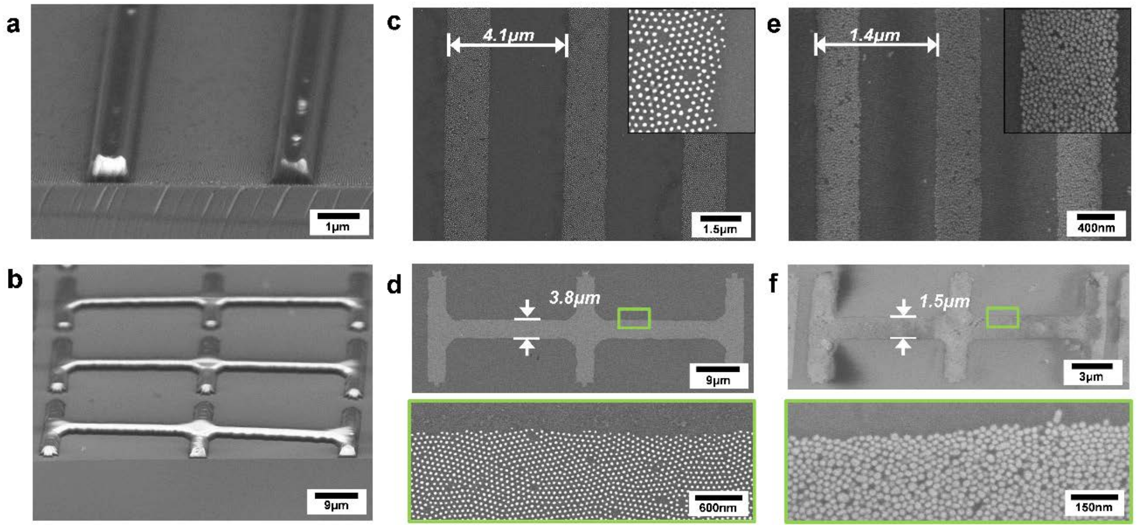

Supplementary Figure 15 | Area-selective metallic nanoparticle ensembles fabrication. **a**, **b**, SEM images of line (**a**) & complex photoresist (PR) micro-pattern (**b**) on silicon substrate with Au nanoparticle array. **c**, **d**, SEM images of lithographically defined line (**c**) & complex pattern (**d**) with Au nanoparticle array after selective wet removal of Au and lift-off of PR. **e**, **f**, SEM images after pattern shrinkage of **c**, **d**.

Supplementary Discussion

We measured the anisotropic scattering of metasurface with respect to the incident angle using FDTD simulations and compared it to that of a thin film made of a uniform effective medium (a uniaxial crystal). The silver nanoparticle structure with shrunken inter-particle distance was used as the metasurface. Gradually changing the angle of incidence, we compared the complex transmission and reflection coefficients calculated from the simulation of the metasurface to the coefficients extracted from an effective anisotropic thin film with a transfer matrix method, in off-resonance regime (at 1000 nm wavelength).

In Supplementary Fig. 10, one can find that the optical properties of the metasurface are very similar to a uniform effective material for all incident angles considered and for both p- and s-polarizations. (The parameters for the effective material were $\varepsilon_x = \varepsilon_y = 13.7 + 0.4i$ and $\varepsilon_z = 3.8 + 0.7i$.) Since a general illumination with arbitrary polarization and spatial patterns can be decomposed into plane waves with p- and s-polarizations, this result indicates that the metasurface can be modelled with good accuracy as a thin film made of a uniform uniaxial crystal when its linear optical properties are concerned.
